# Development and Optimization of a Rapid Colorimetric Membrane Immunoassay for *Porphyromonas gingivalis*

**DOI:** 10.4014/jmb.2103.03029

**Published:** 2021-04-05

**Authors:** Jiyon Lee, Myoung-Kwon Choi, Jinju Kim, SeChul Chun, Hong-Gyum Kim, HoSung Lee, JinSoo Kim, Dongwook Lee, Seung-Hyun Han, Do-Young Yoon

**Affiliations:** 1 Department of Bioscience and Biotechnology, Konkuk University, Seoul 05029, Republic of Korea; 2 Department of Environmental Health Science, Konkuk University, Seoul 0509, Republic of Korea; 3 STARGO Inc., Bucheon 14727, Republic of Korea; 4 Department of Oral Microbiology and Immunology, DRI and BK21 Plus Program, School of Dentistry, Seoul National University, 08826, Republic of Korea

**Keywords:** *Porphyromonas gingivalis*, periodontitis, visual membrane immunoassay

## Abstract

*Porphyromonas gingivalis* (*P. gingivalis*) is a major bacterial pathogen that causes periodontitis, a chronic inflammatory disease of tissues around the teeth. Periodontitis is known to be related to other diseases, such as oral cancer, Alzheimer’s disease, and rheumatism. Thus, a precise and sensitive test to detect *P. gingivalis* is necessary for the early diagnosis of periodontitis. The objective of this study was to optimize a rapid visual detection system for *P. gingivalis*. First, we performed a visual membrane immunoassay using 3,3′,5,5′-tetramethylbenzidine (TMB; blue) and coating and detection antibodies that could bind to the host laboratory strain, ATCC 33277. Antibodies against the *P. gingivalis* surface adhesion molecules RgpB (arginine proteinase) and Kgp (lysine proteinase) were determined to be the most specific coating and detection antibodies, respectively. Using these two selected antibodies, the streptavidin-horseradish peroxidase (HRP) reaction was performed using a nitrocellulose membrane and visualized with a detection range of 103–105 bacterial cells/ml following incubation for 15 min. These selected conditions were applied to test other oral bacteria, and the results showed that *P. gingivalis* could be detected without crossreactivity to other bacteria, including *Streptococcus mutans* and *Escherichia fergusonii*. Furthermore, three clinical strains of *P. gingivalis*, KCOM 2880, KCOM 2803, and KCOM 3190, were also recognized using this optimized enzyme immunoassay (EIA) system. To conclude, we established optimized conditions for *P. gingivalis* detection with specificity, accuracy, and sensitivity. These results could be utilized to manufacture economical and rapid detection kits for *P. gingivalis*.

## Introduction

Periodontitis, a common microbial disease, is a major cause of immunological reaction restriction and tooth loss. According to a recent report from the National Health and Nutrition Examination Survey, an estimated 42%of adults in the US above 30 years of age have periodontitis, with 7.8% having severe periodontitis [1]. Furthermore, the strong correlation between periodontitis and fatal systemic diseases such as oral cancer [2], Alzheimer’s disease [3], and rheumatism [4] is well known. One of the principal pathogenic bacteria causing periodontitis is *Porphyromonas gingivalis*, a gram-negative oral anaerobe present in periodontal connective tissues [5]. *P. gingivalis* induces inflammatory diseases and destroys the tissues supporting the teeth. In our study, we used the *P. gingivalis* laboratory strain ATCC 33277 as well as the clinical strains KCOM2800, KCOM2803, and KCOM3190.

*P. gingivalis* produces many virulence factors which are the main causative agents of periodontal diseases. Among them, arginine- and lysine-specific cysteine proteinases in extracellular membrane adhesion molecules are associated with the incapacitation of the host defense system and the development of inflammation on the gingiva [6]. Proteinases, referred to as Arg-gingipain B (RgpB) and Lys-gingipain (Kgp), are necessary for the growth and proliferation of *P. gingivalis* by obtaining nutrients from the environment [7]. Furthermore, the critical functions of RgpB and Kgp as proteolytic enzymes are the dismantling of host proteins in a broad range. Therefore, they may be key markers for the periodontal pathogenesis of *P. gingivalis*. Thus, we also used antibodies against RgpB and Kgp to detect *P. gingivalis* in the visual membrane immunoassay.

The visual membrane immunoassay is widely used in research to develop kits for disease diagnosis [8-10]. It is a sensitive and rapid on-site method to detect analytes using antibodies and to produce visually readable results on the membrane. The analyte is captured with a membrane-coating antibody and a biotinylated antibody in a sandwich format. The biotinylated antibodies are then conjugated with horseradish peroxidase (HRP). When HRP is activated by the insoluble substrate 3,3′,5,5′-tetramethylbenzidine (TMB; blue), a blue reaction becomes visible on the membrane. In this assay, *P. gingivalis* was used as an analyte for early diagnosis of periodontitis, and antibodies specific to the proteases RgpB and Kgp were used to quantify *P. gingivalis*. We optimized many different conditions of the visual membrane immunoassay for *P. gingivalis*, including the antibodies and three types of membranes (nitrocellulose, polyvinylidene difluoride [PVDF], and hybond nylon). In addition, the reaction times were optimized to a point where the color on the membrane could appear with a meaningful number of *P. gingivalis* cells, at least 10^3^ cells/ml, to make the detection system as rapid and sensitive as possible.

When nitrocellulose membranes pre-coated with RgpB antibody are used, *P. gingivalis* can be detected by the naked eye at a limit of detection (LOD) of 10^3^ cells/ml, which is more sensitive than the LODs of other in situ techniques such as lateral flow immunoassay systems. In addition, these selected conditions can be used to develop other in situ diagnostic kits for periodontitis.

## Materials and Methods

### Bacterial Strains and Reagents

*P. gingivalis* ATCC 33277 (laboratory strain), KCOM2800, KCOM2803, and KCOM3190 (clinical strains) and *E. fergusonii* were supplied by Professor SH Han (Department of Oral Microbiology and Immunology, School of Dentistry, Seoul National University). *S. mutans* was supplied by Professor SC Chun (Department of Environmental Health Science, Konkuk University). BD Gaspak EZ Pouch systems were purchased from BD Biosciences (USA). Antibodies specific to RgpB and Kgp were obtained from Cusabio (USA), and used as coating antibodies. Biotinylated antibodies specific to RgpB and Kgp were used as capture antibodies. The nitrocellulose membrane (pore size: 0.45 μm) was obtained from Whatman (Maidstone, England). Streptavidin-HRP was purchased from R&D Systems (USA).

### Cell Culture

Bacterial cells were cultured in brain heart infusion (BHI) (BD Biosciences) containing broth (37 g/l) with hemin (Cayman Chemical, USA) (5 μg/ml) and menadione (Sigma-Aldrich, USA) (5 μg/ml). Cells were incubated at 37°C in an atmosphere of 10% CO^2^, 10% H_2_, and 80% N_2_.

### Cell Counting by Measuring Optical Density

Cells were suspended in BHI-hemin/menadione medium and serially diluted at least 13 folds. The absorption of each dilution was measured at an optical density (OD) of 600 nm using a UV spectrophotometer. The cells were counted microscopically (×1,000) using a counting chamber. A standard curve of the number of cells (10^6^ cells/ml) counted against an optical density of 600 nm was constructed using multiple samples. When the R^2^ value of the standard curve was over 0.95, a linear regression equation was obtained: Y = 9976.1X + 16.723 (R^2^ = 0.9994).

### Visual Membrane Enzyme Immunoassay (EIA)

The membranes were placed in a solution of the coating antibody (2 μg/ml) for 2 h at room temperature (RT) and washed three times with tris-buffered saline (TBS) containing 0.5% Tween-20 (TBST) (2.7 M NaCl, 1 M Tris-HCl, 53.65 mM KCl, and 0.1% Tween-20; pH 7.4). After nonspecific binding was blocked by soaking the membranes in phosphate-buffered saline (PBS) containing 3% bovine serum albumin (BSA) for 1 h and washing three times with TBST, the antibody-coated membranes were cut into small pieces (larger than 0.5 × 0.5 cm). The pieces were placed in a 24-well plate containing a mixture of *P. gingivalis* cells (10^3^, 10^4^, and 10^5^ cells/ml) and biotinylated antibody (20 ng/ml) in TBST. After 10 min of incubation at RT, the membranes were washed three times with TBST and placed in streptavidin (SA)-HRP in PBS for 15 min. The membranes were then washed three times with TBST, followed by soaking in TMB substrate solution for 5 min. Finally, the membranes were washed with distilled water and dried.

### Statistical Analysis

Experiments were performed in triplicate. The color staining the membrane was quantified using ImageJ software version 1.5 [11]. Data are presented as mean ± SD and analyzed by Student’s t-test with one-way ANOVA followed by Tukey’s honestly significant difference (HSD) test. Statistical significance was set at *p* < 0.05.

## Results and Discussion

### Optimization of Membrane, Reaction Time, and Antibodies for Rapid Visual Detection of *P. gingivalis*

**Fig. 1:**
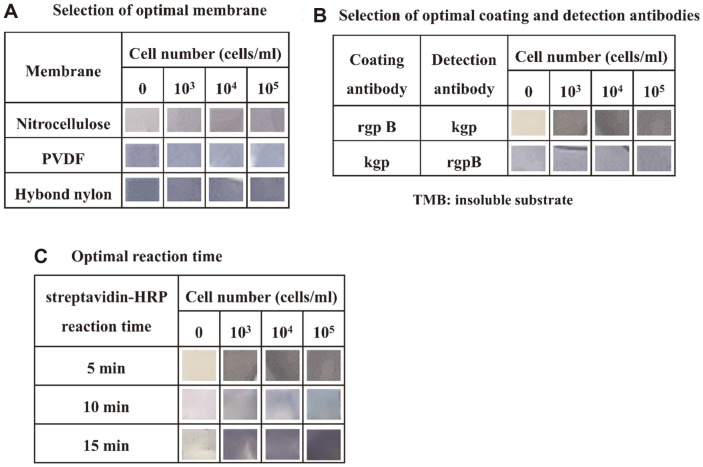
Optimization of membrane, reaction time, and antibodies for a rapid visual detection system of *P. gingivalis* ATCC 33277. **A**, Selection of optimal membrane; **B**, Selection of optimal coating and detection antibodies; and **C**, Optimal reaction time. Various membranes (nitrocellulose membrane, PVDF, Hybond nylon), various reaction times, and combination of coating and detection antibodies were tested for a rapid visual detection system of *P. gingivalis* ATCC 33277.

**Fig. 2:**
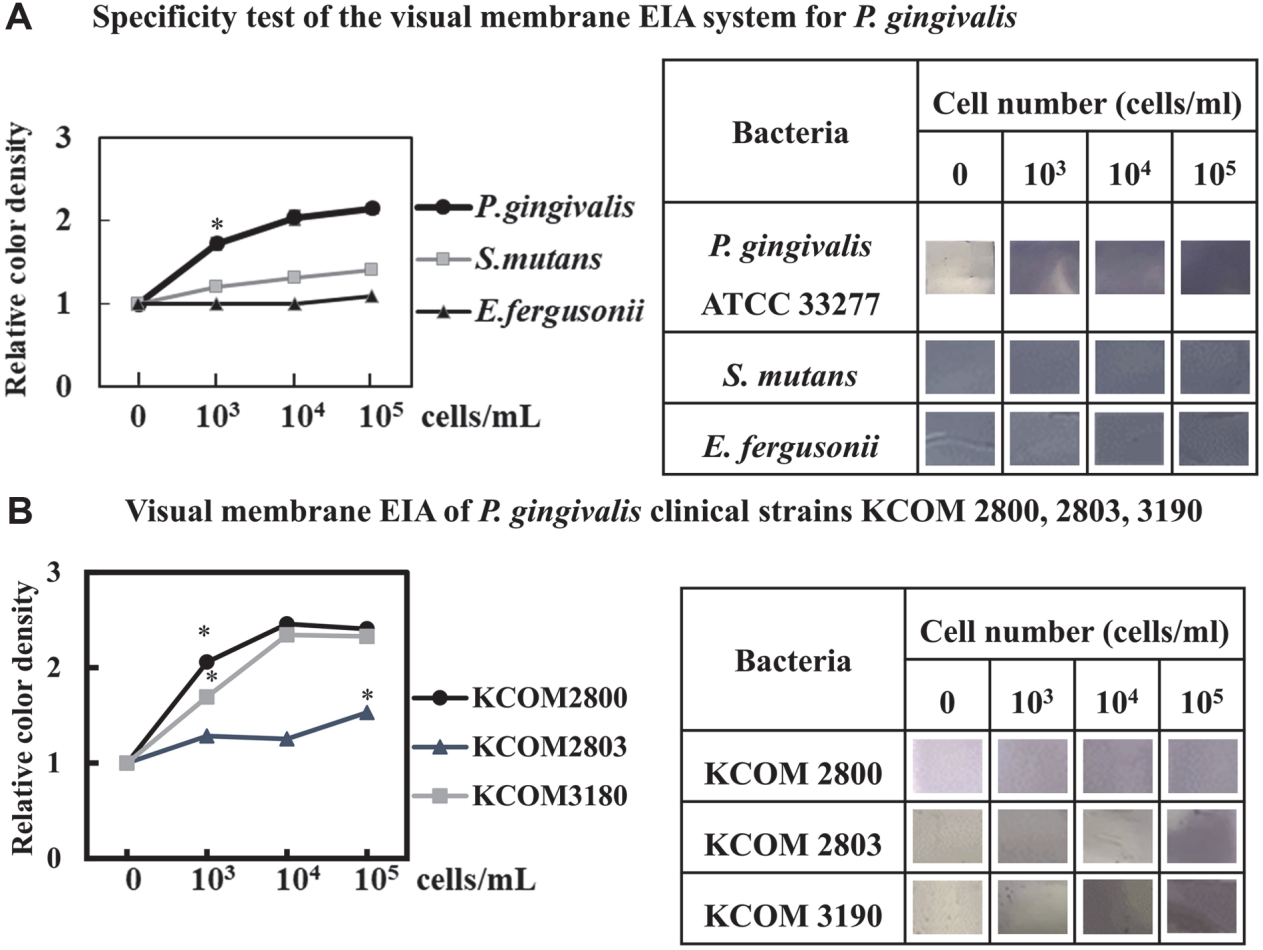
Evaluation of a visual membrane immunoassay for *P. gingivalis* using an RgpB antibody and a biotinylated Kgp antibody. RgpB antibody and biotinylated Kgp antibody were used as coating and capturing antibody, respectively, as in Fig. 1B. **A**, Sensitivity test of a visual membrane EIA system for *P. gingivalis*. **B**, Visual membrane EIA of *P. gingivalis* clinical strains KCOM 2800, 2803, 3190. Visual membrane EIA system for *P. gingivalis* was also confirmed to detect other strains such as *S. mutans* and *E. fergusonii* or not. Results represent the mean ± SD of three experiments (vs. negative control, *p < 0.05).

To optimize the conditions for the visual membrane EIA, various membranes were coated with the RgpB antibody. The membranes were incubated with *P. gingivalis* cells and biotinylated Kgp antibodies in a 24-well plate. After treatment with streptavidin (SA)-HRP for 15 min followed by soaking in the TMB substrate solution for 5 min, 10^3^/ml of *P. gingivalis* cells could be discriminated from the nitrocellulose membrane (Figs. 1A and 2A). There were neither color differences between PVDF and Hybond nylon, and nor were there color differences when the Kgp and Rgp antibodies were used as coating and detection antibodies, respectively (Fig. 1B). These results revealed that the optimum reaction conditions were as follows: nitrocellulose membrane, antibody coated with RgpB, antibody biotinylated with Kgp, SA-HRP for 15 min, and use of a TMB substrate solution for 5 min. The results of the visual membrane EIA largely depend on the choice of the matrix as a solid support. The matrix should be a neutral polymer with minimal nonspecific interaction with biomolecules and the components of the staining solution and should be able to absorb quantitatively the final insoluble product of the enzyme reaction [8]. During immobilization and under reaction conditions, the mechanical strength and chemical stability of the matrix are very important for selection. Whatman nitrocellulose paper satisfied the above requirements compared to PVDF and Hybond nylon and was selected for use as a matrix in this visual membrane EIA. o-Phenylene diamine (OPD) and diaminobenzidine (DAB) have been used for the colorimetric determination of HRP. During chemical reactions, OPD and DAB show mutagenic and carcinogenic properties [12, 13]. The chromogenic substrate TMB is preferred over DAB or OPD because it lacks mutagenic and carcinogenic properties and is insensitive to light when prepared in dimethyl sulfoxide (DMSO) [14]. Moreover, the color developed was stable for longer than that of OPD and DAB. Thus, in this study, TMB was used in the membrane immunoassay.

### Evaluation of Visual Membrane EIA for *P. gingivalis*, *S. mutans* and *E. fergusonii* Using RgpB and Biotinylated Kgp Antibodies

Our optimized visual membrane EIA system for *P. gingivalis* was tested to elucidate whether it would detect other strains. The system did not discriminate colors of other *S. mutans* strains or *E. fergusonii* compared to negative control (Fig. 2A). Furthermore, the visual membrane EIA system successfully detected all three of our tested *P. gingivalis* clinical strains, KCOM 2800, 2803, and 3190 (Fig. 2B).

### Overview of Visual Membrane EIA for *P. gingivalis* Using Antibodies Against RgpB, Biotinylated Kgp, and Nitrocellulose Membrane

**Fig. 3:**
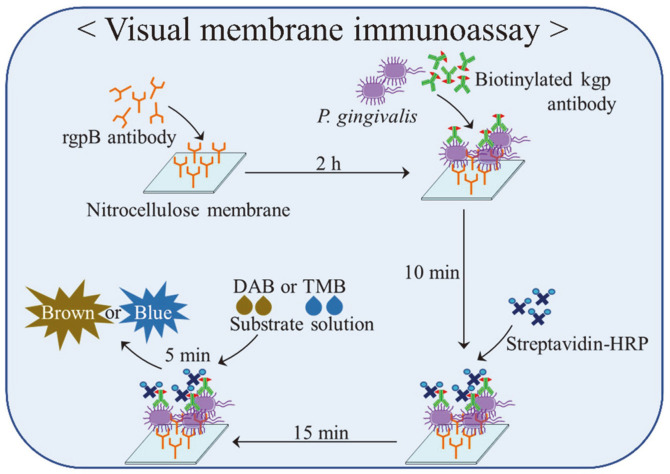
Overview of a visual membrane immunoassay for *P. gingivalis* using an RgpB antibody, biotinylated Kgp antibody, and nitrocellulose membrane. The membranes were coated with an RgpB antibody (2 μg/ml), followed by nonspecific binding with 3% BSA and cut into small pieces (larger than 0.5 × 0.5 cm). The pieces were placed in a 24-well plate containing a mixture of *P. gingivalis* cells and a biotinylated Kgp antibody (20 ng/ml). The membrane was washed, placed in streptavidin (SA)-HRP for 15 min, followed by soaking in the TMB substrate solution for 5 min. The membrane was washed with distilled water and dried.

All steps of the immunoassay procedure were performed on an "immuno" membrane immobilized with antibodies as outlined in Fig. 3. When the RgpB antibody-precoated "immuno" membranes capture *P. gingivalis*, biotinylated Kgp antibody and streptavidin (SA)-HRP, a change in color was noted after treating with TMB, indicating that *P. gingivalis* cells could be semi-quantified (Figs. 1 and 2). This visual membrane EIA system was optimized as follows: two antibodies against cell surface molecules were selected to visualize the streptavidin-HRP reaction using a nitrocellulose membrane, with a detection range of 10^3^–10^5^ bacteria cells/ml. Furthermore, these selected conditions were also applied to test other oral bacteria. Three clinical strains of *P. gingivalis*, KCOM 2880, KCOM 2803, and KCOM 3190, were recognized using this optimized visual membrane EIA system (Fig. 2B). Overall, based on the limit of detection (LOD) of this membrane EIA, 10^3^/ml of *P. gingivalis* cells could be detected and there was no cross-reactivity with other oral bacteria such as *S. mutans* strains or *E. fergusonii* (*p* < 0.05, compared to the negative control, Fig. 2A).

There are interesting emerging immunochemical analytical techniques for sensitive in situ detection methods. A membrane EIA is cheap, easy to prepare, has sensitivity on *P. gingivalis* with no devices for detection because it can be observed with the naked eye. However, this method takes more time than other in situ techniques like the lateral flow immunoassay, a commercially available detection method that takes 3-5 min, while the PCR takes 90-120 min. In case of our visual membrane EIA, the LOD is 10^3^ cells/ml (Figs. 1 and 2), which is 100 times more sensitive than the LOD of other lateral flow immunoassays (10^5^ cells/ml) and almost the same as those of PCRs [15, 16]. The multiplex lateral flow immunoassay has been widely used as an in situ detection method using colloidal gold nanoparticles, which are expensive [17]. In situ screening tests require a simple and rapid instrument-independent procedure. These “express” tests can detect a few specific target molecules on cells (bacteria, virus, or cancer) qualitatively or semi-quantitatively and utilize reagents immobilized on the nitrocellulose membranes of porous carriers [18]. In conclusion, these types of visual membrane EIAs can be applied to the development of an in situ, simple and rapid immunochromatographic strip test.

## References

[1] Eke PI, Thornton-Evans GO, Wei L, Borgnakke WS, Dye BA, Genco RJ (2018). Periodontitis in US adults: national health and nutrition examination survey 2009-2014. J. Am. Dent. Assoc..

[2] Fitzpatrick SG, Katz J (2010). The association between periodontal disease and cancer: a review of the literature. J. Dent..

[3] Kamer AR, Pirraglia E, Tsui W, Rusinek H, Vallabhajosula S, Mosconi L (2015). Periodontal disease associates with higher brain amyloid load in normal elderly. Neurobiol. Aging.

[4] de Pablo P, Chapple IL, Buckley CD, Dietrich T (2009). Periodontitis in systemic rheumatic diseases. Nat. Rev. Rheumatol..

[5] Mysak J, Podzimek S, Sommerova P, Lyuya-Mi Y, Bartova J, Janatova T (2014). *Porphyromonas gingivalis*: major periodontopathic pathogen overview. J. Immunol. Res..

[6] Kadowaki T, Nakayama K, Okamoto K, Abe N, Baba A, Shi Y (2000). *Porphyromonas gingivalis* proteinases as virulence determinants in progression of periodontal diseases. J. Biochem..

[7] Abe N, Baba A, Takii R, Nakayama K, Kamaguchi A, Shibata Y (2004). Roles of Arg- and Lys-gingipains in coaggregation of *Porphyromonas gingivalis*: identification of its responsible molecules in translation products of rgpA, kgp, and hagA genes. Biol. Chem..

[8] Yoon DY, Song EY, Kwon DH, Choi MJ, Byun SM, Choe IS (1995). Use of progesterone-3(O-carboxymethyl oxime)-horseradish peroxidase in a sensitive microtitre-plate EIA and its application to a visual membrane EIA of progesterone. J. Immunoassay.

[9] Tominaga T (2018). Rapid detection of *Klebsiella pneumoniae*, Klebsiella oxytoca, Raoultella ornithinolytica and other related bacteria in food by lateral-flow test strip immunoassays. J. Microbiol. Methods.

[10] Lathwal S, Sikes HD (2016). Assessment of colorimetric amplification methods in a paper-based immunoassay for diagnosis of malaria. Lab Chip..

[11] Schneider CA, Rasband WS, Eliceiri KW (2012). NIH Image to ImageJ: 25 years of image analysis. Nat. Methods.

[12] Voogd CE, Van der Stel JJ, Jacobs JJ (1980). On the mutagenic action of some enzyme immunoassay substrates. J. Immunol. Methods.

[13] Bos ES, van der Doelen AA, van Rooy N, Schuurs AH (1981). 3,3',5,5' - Tetramethylbenzidine as an Ames test negative chromogen for horse-radish peroxidase in enzyme-immunoassay. J. Immunoassay.

[14] Porstmann B, Evers U, Nugel E, Schmechta H (1991). [Tetramethylbenzidine--a chromogenic substrate for peroxidase in enzyme immunoassay]. Z Med. Lab. Diagn..

[15] O'Brien-Simpson NM, Burgess K, Lenzo JC, Brammar GC, Darby IB, Reynolds EC (2017). Rapid chair-side test for detection of *Porphyromonas gingivalis*. J. Dent. Res..

[16] Gu BL, Qi YJ, Kong JY, Li ZT, Wang JP, Yuan X (2020). An evaluation of direct PCR assays for the detection and quantification of *Porphyromonas gingivalis*. Epidemiol. Infect..

[17] Foubert A, Beloglazova NV, De Saeger S (2017). Comparative study of colloidal gold and quantum dots as labels for multiplex screening tests for multi-mycotoxin detection. Anal. Chim. Acta.

[18] Kang JH, Kwon DH, Chung TW, Kim YD, Lee HG, Kim JW (2007). Development of a simple and rapid immunochromatographic strip test for diarrhea-causative porcine rotavirus in swine stool. J. Virol. Methods.

